# Pharmacokinetics and Metabolism of Cyadox and Its Main Metabolites in Beagle Dogs Following Oral, Intramuscular, and Intravenous Administration

**DOI:** 10.3389/fphar.2016.00236

**Published:** 2016-08-03

**Authors:** Adeel Sattar, Shuyu Xie, Lingli Huang, Zahid Iqbal, Wei Qu, Muhammad A. Shabbir, Yuanhu Pan, Hafiz I. Hussain, Dongmei Chen, Yanfei Tao, Zhenli Liu, Mujahid Iqbal, Zonghui Yuan

**Affiliations:** ^1^National Reference Laboratory of Veterinary Drug Residues, Huazhong Agricultural UniversityWuhan, China; ^2^MOA Laboratory for Risk Assessment of Quality and Safety of Livestock and Poultry Products, Huazhong Agricultural UniversityWuhan, China; ^3^MAO Key Laboratory for Detection of Veterinary Drug Residues, Huazhong Agricultural UniversityWuhan, China

**Keywords:** cyadox, pharmacokinetics, metabolism, beagle dogs, metabolites

## Abstract

Cyadox (Cyx) is an antibacterial drug of the quinoxaline group that exerts markedly lower toxicity in animals, compared to its congeners. Here, the pharmacokinetics and metabolism of Cyx after oral (PO), intramuscular (IM), and intravenous (IV) routes of administration were studied to establish safety criteria for the clinical use of Cyx in animals. Six beagle dogs (3 males, 3 females) were administered Cyx through PO (40 mg kg^−1^ b.w.), IM (10 mg kg^−1^ b.w.), and IV (10 mg kg^−1^ b.w.) routes with a washout period of 2 weeks in a crossover design. Highly sensitive high-performance liquid chromatography with ultraviolet detection (HPLC-UV) was employed for determination of Cyx and its main metabolites, 1, 4-bisdesoxycyadox (Cy1), cyadox-1-monoxide (Cy2), N-(quinoxaline-2-methyl)-cyanide acetyl hydrazine (Cy4), and quinoxaline-2-carboxylic acid (Cy6) in plasma, urine and feces of dogs. The oral bioavailability of Cyx was 4.75%, suggesting first-pass effect in dogs. The concentration vs. time profile in plasma after PO administration indicates that Cyx is rapidly dissociated into its metabolites and eliminated from plasma earlier, compared to its metabolites. The areas under the curve (AUC) of Cyx after PO, IM and IV administration were 1.22 h × μg mL^−1^, 6.3 h × μg mL^−1^, and 6.66 h × μg mL^−1^, while mean resident times (MRT) were 7.32, 3.58 and 0.556 h, respectively. Total recovery of Cyx and its metabolites was >60% with each administration route. In feces, 48.83% drug was recovered after PO administration, while 18.15% and 17.11% after IM and IV injections, respectively, suggesting renal clearance as the major route of excretion with IM and IV administration and feces as the major route with PO delivery. Our comprehensive evaluation of Cyx has uncovered detailed information that should facilitate its judicious use in animals by improving understanding of its pharmacology.

## Introduction

Quinoxaline 1, 4-di-N-oxide (QdNO) derivatives are effective synthetic antibacterial agents used worldwide since 1970 to treat several gram-positive and gram-negative bacterial infections in animals (Wang et al., 2015a,b; Cheng et al., 2016). Olaquindox, mequindox, and carbadox are the well-known members of this group, but have been banned or strictly limited to use due to their potential toxicities in animals. Cyadox (Cyx), 2-formylquinoxaline-N^1^, N^4^-dioxide cyanocetylhydrazone, is a novel derivative of QdNO, and a promising antibacterial agent believed to be safer for use than its congeners (Fan et al., 2000; Fang et al., 2006; Wang et al., 2015c). It has been characterized as an effective broad-spectrum antibacterial agent against the majority of pathogenic bacteria (including *Staphylococcus, Pasturella*, and *Salmonella* spps) in animals (Fan et al., 2000; Huang et al., 2002; Wang et al., 2005). Cyx has been recommended as the drug of choice to prevent *Escherichia coli* infection in pigs and chickens owing to the lack of toxic effects (Wang et al., 2005; Ding et al., 2006a,b). Dogs are generally more prone to these types of bacterial infections (Angus et al., 1997; Greene, 2013; Lowden et al., 2015). The findings to date support the efficacy of Cyx as a safe and effective quinoxaline-based antibacterial agent in different animal species, including canines.

During the drug development process, failures are often attributed due to adverse pharmacokinetic properties of the tested compounds. Bioavailability and desirable half-life are the key parameters for consideration to establish the success of newly formed drugs. Accurate assessment of pharmacokinetics and affecting factors is necessary for effective drug design. Therefore, pharmacokinetic and metabolic profiles should be taken into consideration to facilitate rational drug design (Lin and Lu, 1997). Dogs have long been used as a model in drug development and discovery research due to their anatomical and physiological resemblance with other mammals (Smith, 1991; Khanna et al., 2006; Rowell et al., 2011) and thus regarded as optimal choice for evaluation of new drugs, in view of the similarities between the pharmacokinetic and metabolism profiles (Smith, 1991; Lin, 1995; Chiou et al., 2000; Weinz et al., 2009). Many studies have been conducted using dogs as a surrogate for humans (Sande and Johnson, 1975; Rossi et al., 2004; Knapp et al., 2014; Lin et al., 2015; Piras et al., 2015). Evaluation of metabolism, distribution and excretion are of vital importance for determining the fate of a particular drug within the body. Numerous *in vitro* and *in vivo* studies have demonstrated that Cyx is extensively metabolized through different pathways and many kinds of metabolites identified in pigs, chicken, carp, and rats (Liu et al., 2009; Xu et al., 2011; Huang et al., 2015). Furthermore, some approaches for the quantification of Cyx and its various metabolites in animal tissues have been described (Huang et al., 2008; Cheng et al., 2012; Yan et al., 2012). Tissue depletion and pharmacokinetics of Cyx and its main metabolites following oral administration in pigs were recently investigated (Li et al., 2013; Zhao et al., 2013). However, comprehensive pharmacokinetics, distribution and depletion profiles of Cyx in rodents and non-rodents are yet to be completely established. To date, a small number of studies have focused on the pharmacokinetic properties of Cyx without considering its main metabolites (Qiu, 2003; Yin, 2003; Huang, 2005). Given that Cyx is metabolized extensively and rapidly in the body (Liu et al., 2009; Xu et al., 2011), the main metabolites of Cyx must be well thought-out during metabolism and pharmacokinetic studies for clarification of the fate of the drug. Previous studies conducted by the team of National Basic Research Program of China (973 Program, 2009CB118800) and other research groups have disclosed that 1, 4-bisdesoxycyadox (Cy1), cyadox-1-monoxide (Cy2), N-(quinoxaline-2-methyl)-cyanide acetyl hydrazine (Cy4) and quinoxaline-2-carboxylic acid (Cy6) are the main metabolites of Cyx in different animals (Zhao et al., 2013; Huang et al., 2015). Information on the metabolism and pharmacokinetic properties of Cyx and its main metabolites is therefore critical for comprehensive assessment of the fate of the drug in animals to ensure its judicious use as an antibacterial.

Cyx is less toxic in non-rodents, compared to other QdNO derivatives (Wang et al., 2015c). To our knowledge, no relevant studies on Cyx to establish safety criteria have been reported in non-rodents. In view of the physiological similarities of dogs with humans and other animals, the present study aimed to explore the metabolism and pharmacokinetic profiles of Cyx and its main metabolites (Cy1, Cy2, Cy4, and Cy6) in beagle dogs following oral (PO), intramuscular (IM), and intravenous (IV) administration using high-performance liquid chromatography with ultraviolet (HPLC-UV) detection. Our results should be useful for the assessment of efficacy, safety and effective dosage regimens of Cyx for clinical use.

## Materials and methods

### Chemicals

Cyx (≥98%) and its four metabolites Cy1, Cy2, Cy4, and Cy6 were synthesized at the Veterinary Institute of Pharmaceuticals in Huazhong Agricultural University (Wuhan, PR China). HPLC-grade acetonitrile (ACN) and methanol (MeOH) were purchased from Fisher Chemicals Co. (New Jersey, USA). Oasis HLB cartridges (3 mL, 60 mg) were acquired from Waters (Milford, MA, USA). Polyvinylpyrrolidone K30 (PVP), sodium hydroxide (NaOH), hydrochloric acid (HCl), dimethyl sulfoxide (DMSO), sodium chloride (NaCl), and trichloromethane (TCM) were obtained from Sinopharm Chemical Reagent Co., LTD (Shanghai, PR China). Water was purified using the Milli-Q water purification system (Milli-Q Co., Ltd, France). All other chemicals and reagents used were of high analytical grade.

### Solution preparation

Standard stock solutions of Cyx, Cy1, Cy2, Cy4, and Cy6 were prepared individually by dissolving in DMSO and stored at −20°C in the dark. All metabolites were mixed together and different concentrations (10–100 μg mL^−1^) prepared using MeOH before storage at 4°C for further use.

### Formulation preparation

Cyx suspension (80 mg mL^−1^) was prepared for PO administration in dogs by dissolving an appropriate amount into 0.5% sodium carboxymethyl cellulose aqueous solution. Due to the likelihood of increased toxicity of Cyx upon dissolving in DMSO, suitable suspension for IM and IV routes of administration was generated by recrystallization based on acid-base neutralization for safe use in animals. Initially, a number of solutions were prepared in advance, including NaOH (12 mol L^−1^), 1% PVP and HCl (12 mol L^−1^). The NaOH solution (15 mL) was poured into a 200 mL volumetric flask containing 20 mL 1% PVP stabilizer under stirring using a thermostat magnetic stirrer (90-1, Shanghai HuXi analysis instrument factory Co., Ltd., Shanghai, PR China) and 1 g of Cyx subsequently added. After Cyx was completely dissolved, 15 mL HCl was dissolved for acid-base neutralization. A uniform suspension of Cyx (50 mL) was obtained at a concentration of 20 mg mL^−1^. The viability of the stock solution was determined using a UV spectrophotometer before IM and IV injection into dogs.

### Animals

Three male and three female healthy Beagle dogs 5–6 months of age with uniform weights (9–10 kg) were purchased from the Experimental Animal Center of Tongji University (Wuhan, PR China). Animals were maintained under conditions of ambient temperature (22 ± 2°C) with 45–65% relative humidity. Air ventilation was provided every 30 min. Dogs were fed 350 g certified commercial diet (Ke Ao Xie Li Co. Ltd., Beijing, PR China) at a fixed time per day, with tap water provided *ad libitum* throughout the experiment. Each dog was kept in an isolated steel cage (1000 × 10,000 × 900 mm) with a specific ear tag number 2 weeks before the start of the experiment for acclimatization. Identification numbers were also attached to every cage. All dogs enjoyed outside walk at least once a week. This experiment was performed in accordance with NIH publication 85–23 “Guide for the Care and Use of Laboratory Animals” (NRC, 1996) and approved by the Ethical Committee of the Faculty of Veterinary Medicine at Huazhong Agricultural University.

### Dose administration and sampling

A randomized crossover design with a 2-week washout period was employed. PO administration was achieved through gavage at a dose rate of 40 mg kg^−1^ b.w., and the blood (1 mL) collected from the cephalic vein in heparin-containing tubes before and after 0.25, 0.50, 1, 2, 3, 4, 5, 6, 7, 8, 10, 12, 14, 16, 24, 36, and 48 h of administration. IM administration was achieved through injection into the quadricep muscle within the thigh region at a dose rate of 10 mg kg^−1^ b.w., and blood (1 mL) collected in heparin-containing tubes before and after 0.25, 0.5, 0.75, 1, 2, 3, 4, 5, 6, 7, 8, 10, 12, 16 and 24 h of injection. For IV administration, the drug was injected into the cephalic vein at a dose rate of 10 mg kg^−1^ b.w., and blood (1 mL) collected in heparin-containing tubes before and after 0.033, 0.083, 0.167, 0.25, 0.333, 0.50, 0.75, 1, 1.5, 2, 3, 4, 5, 6, 7 and 8 h of injection. All blood samples were centrifuged at 5000 × g for 15 min at 4°C. Plasma was separated and stored at −20°C until UV-HPLC analysis. Furthermore, the total amount of urine and feces were collected in ice bags before and after 12 and 24 h, followed by intermittent collection from days 1 to 14 after each administration. The total volumes and weights of each sample were measured and homogenized. An appropriate quantity of each sample was stored at −20°C until UV-HPLC analysis.

### Sample treatment

#### Blood

An aliquot of 0.3 mL plasma from each time-point was added into a polypropylene centrifuge tube. Deproteinization was performed with 0.3 mL methanol, followed by vigorous vortexing for 3 min. Samples were centrifuged at 10,000 × g for 10 min at 4°C and supernatant fractions filtered through a 0.22 μm microbore cellulose membrane. A 200 μL aliquot of each sample was injected onto the HPLC system.

#### Urine

Urine sample (2 mL) was poured into a 10 mL polypropylene plastic tube after filtration through a 0.22 μm microbore cellulose membrane. A stock solution of 2% metaphosphoric acid was added to the tube until pH was maintained at 5–6, followed by vigorous vortexing for 3 min. The supernatant was loaded onto Oasis HLB cartridge (3 mL, 60 mg; Waters, Milford, MA, USA) which was preconditioned with 6 mL methanol and 6 mL water at a flow rate of 3 mL min^−1^. Next, the cartridge was washed with 6 mL of 5% methanol/water (v/v). The filter was dried by purging an airstream at a flow rate of 10 mL min^−1^ for 4–5 min, and analytes finally eluted with 6 mL methanol. Subsequently, 12 mL TCM was added, followed by vigorous vortexing for 3 min and centrifugation at 8000 × g for 10 min. After removing the aqueous layer, the solution was evaporated to dryness under a nitrogen stream at 40°C. Residues were reconstituted with 2 mL of 65% methanol and centrifuged at 10,000 × g for 8 min. Finally, 1 mL supernatant was filtered through a 0.22 μm microbore cellulose membrane before injection into the UV- HPLC system.

#### Feces

Aliquots of homogenized feces (2 g) were placed in 30 mL polypropylene centrifuge tubes, and mixed with 8 mL of 1% metaphosphoric acid in MeOH/ACN/water (50:20:30, v/v/v). The mixtures were vortexed vigorously for 3 min, ultrasonicated for 10 min at room temperature, and centrifuged at 4000 × g for 10 min. The supernatant was collected and transferred into a separate 30 mL centrifuge tube. Yet again the same extraction procedure was repeated. The two supernatants were combined and dried under a nitrogen stream at 40°C. To remove fat from the sample, 6 mL n-hexane was added into the remaining solution, followed by vortexing for 3 min and centrifugation at 8000 × g for 10 min. To confiscate proteins from the sample, 2 mL of 10% zinc acetate was added to the extract, vortexed for 3 min, and centrifuged at 10,000 × g for 10 min, followed by separation of the resultant supernatant which was loaded onto a HLB cartridge. The cleanup procedure described above was similarly adopted for HLB. After elution, 6 mL of water was added to the eluted sample. Next, 12 mL TCM was added, vortexed for 3 min, and after centrifugation at 10,000 × g for 10 min at 4°C, the sample was dried under a nitrogen stream at 40°C. Dried residues were reconstituted with 2 mL of 65% methanol, vortexed for 1 min and centrifuged at 12500 × g for 10 min. The solution was filtered through 0.22 μm nylon Millipore chromatographic filters and a 200 μL aliquot subjected to HPLC for detection of the parent drug and its main metabolites. Total recovery of the drug in urine and feces was calculated with the following formula:

$$\begin{array}{l} X\;=\;\frac{\text{Dc }\times \;V}{Td\;\times \;Wt}\times 100 \end{array}$$

*X* = % drug recovered

Dc = Drug concentration in urine or feces

*V* = Total volume of urine or feces

*Td* = Total dose given to the animal

*Wt* = Weight of animal

### HPLC conditions

All samples (plasma, urine, and feces) were analyzed using HPLC (Waters 2695 series) combined with an ultraviolet detector system with the wavelength set at 320 nm. Separation of chromatographs was achieved using a ZORBAX SB-C 18 column (250 mm × 4.6 mm i.d., 5 μm, Agilent, USA) at a flow rate of 1 mL min^−1^ at 30°C. The gradient elution mode was set for separation of Cyx and its main metabolites, with the mobile phase A containing 0.5% formic acid and mobile phase B containing pure ACN. The gradient profile was set as follows: 0 min, 88% A; 5 min, 80% A; 26 min, 72% A; 26.01 min, 88% A; 30 min, 88% A. The injection volume was 40 μL.

#### Method validation

Limit of detection (LOD) and limit of quantification (LOQ) were calculated based on signal-to-noise ratio of sample presented with 3:1 and 10:1, respectively. To obtain standard curves and linear range, 100 μL of mixed standard solution of Cyx and its metabolites was spiked in samples extracted and purified using the above procedures. The concentrations of Cyx and its metabolites ranged from 0.1 to 6.4 μg mL^−1^ in urine and feces while in plasma, the concentrations ranged from 0.02 to 10.24 μg mL^−1^. Calibration curves were generated using weighted least squares linear regression (1/y) and calculated as y = ax ± b, where y represents the area of analyte and x the concentration of the drug. Correlation coefficient, intercept and slope of each standard curve were calculated. Accuracy and precision were determined by adding three different concentrations (1, 2, and 4 times the LOQ) to blank samples. For the intraday experiment, five sets of each concentration were run while five replicates of three concentrations were examined on five different days for the inter-day experiment. Accuracy was calculated on the basis of absolute recovery by comparing the extracted sample peak area with standard solution peak area. Precision was estimated as relative standard deviation (RSD).

### Data analysis

The non-compartmental model was applied to calculate the pharmacokinetic parameters of Cyx and its metabolites using the WinNonlin software program (5.2 Version, Pharsight Corporation, CA, USA) according to an earlier report (Cutler, 1978; Yamaoka et al., 1978). Maximum concentration in plasma (C_max_) and time to reach at this concentration (T_max_) were calculated from the concentration-time profile. The area under first moment (AUMC_0−∞_) and area under concentration-time curve (AUC_0−∞_) were calculated from zero to the last determined concentration at infinity (Perrier and Mayersohn, 1982). The bioavailability (F) of Cyx after administration via the PO and IM routes was calculated using the formula: *F* = (Dose_IV_× AUC_P.O_)/(Dose_PO_ × AUC_IV_) × 100 and (Dose_IV_× AUC_IM_)/(Dose_IM_ × AUC_IV_) × 100, respectively. All descriptive statistical parameters were presented as means and standard deviation using Microsoft Excel 2010.

## Results

### HPLC method validation

Linearity of the standard mixtures with different concentrations in plasma (0.02–10.24 μg mL^−1^) and urine or feces (0.1–6.4 μg mL^−1^) showed excellent results within the same day as well as on different days. The correlation coefficient values of all five metabolites indicating functional linear relationship at different concentrations were >0.999 across the concentration ranges used for all samples. The standard calibration curves obtained were used to quantify the drug metabolites in plasma, urine and feces of dogs and also to ensure accuracy of method. Furthermore, based on evaluation of background noise with several matrices, no interference peak was detected at the time of retention of the test compound.

The LOD was 0.02 μg mL^−1^ for Cyx, Cy1, Cy2, and Cy6, and 0.04 μg mL^−1^ for Cy4 in dog plasma. In urine and feces, LOD of all analytes was 0.1 μg mL^−1^, based on a signal-to-noise ratio of 3:1. LOQ in dog plasma was 0.05 μg mL^−1^ for all analytes, except Cy4, for which an LOQ of 0.08 μg mL^−1^ was obtained. LOQ of all the analytes was 0.2 μg mL^−1^ in feces and urine with acceptable accuracy and precision at a signal-to-noise ratio of 10:1 (Table 1). The accuracy and precision of this method were calculated at specified concentration range within and at five different days. The mean recovery rate was >80% in plasma and >70% in feces and urine. Inter- and intra-day RSD were < 10%, signifying that the proposed method is accurate and precise for detection of Cyx and its major metabolites (Cy1, Cy2, Cy4, and Cy6) in plasma, urine, and feces.

**Table 1:** **Method validation data of cyadox and its main metabolites in dog plasma, urine, and feces**.

**Sample**	**Drugs**	**Units**	**Linearity concentration range**	**Calibration equation**	**Correlation coefficient (*r*)**	**LOD**	**LOQ**
Plasma	Cy0	μg mL^−1^	0.02–10.24	y = 82570x − 3830.5	0.999	0.02	0.05
	Cy1			y = 122088x + 6241.9	0.9986	0.02	0.05
	Cy2			y = 105565x + 4687.9	0.9996	0.02	0.05
	Cy4			y = 120618x + 6246.9	0.9997	0.04	0.08
	Cy6			y = 55387x − 132.05	0.9999	0.02	0.05
Feces	Cy0	μg g^−1^	0.1–6.4	y = 114609x + 13972	0.9991	0.1	0.2
	Cy1			y = 129119x + 7416.8	0.9993	0.1	0.2
	Cy2			y = 111348x + 1263.5	0.9993	0.1	0.2
	Cy4			y = 117914x − 4828.4	0.9992	0.1	0.2
	Cy6			y = 61228x + 6817.7	0.999	0.1	0.2
Urine	Cy0	μg mL^−1^	0.1–6.4	y = 115770x + 11250	0.9991	0.1	0.2
	Cy1			y = 136069x + 5606.2	0.9997	0.1	0.2
	Cy2			y = 104538x + 6599	0.9996	0.1	0.2
	Cy4			y = 117520x + 5271.3	0.9994	0.1	0.2
	Cy6			y = 90745x + 2336.2	0.9998	0.1	0.2

### Pharmacokinetics of Cyx and its main metabolites

**Figure 1:**
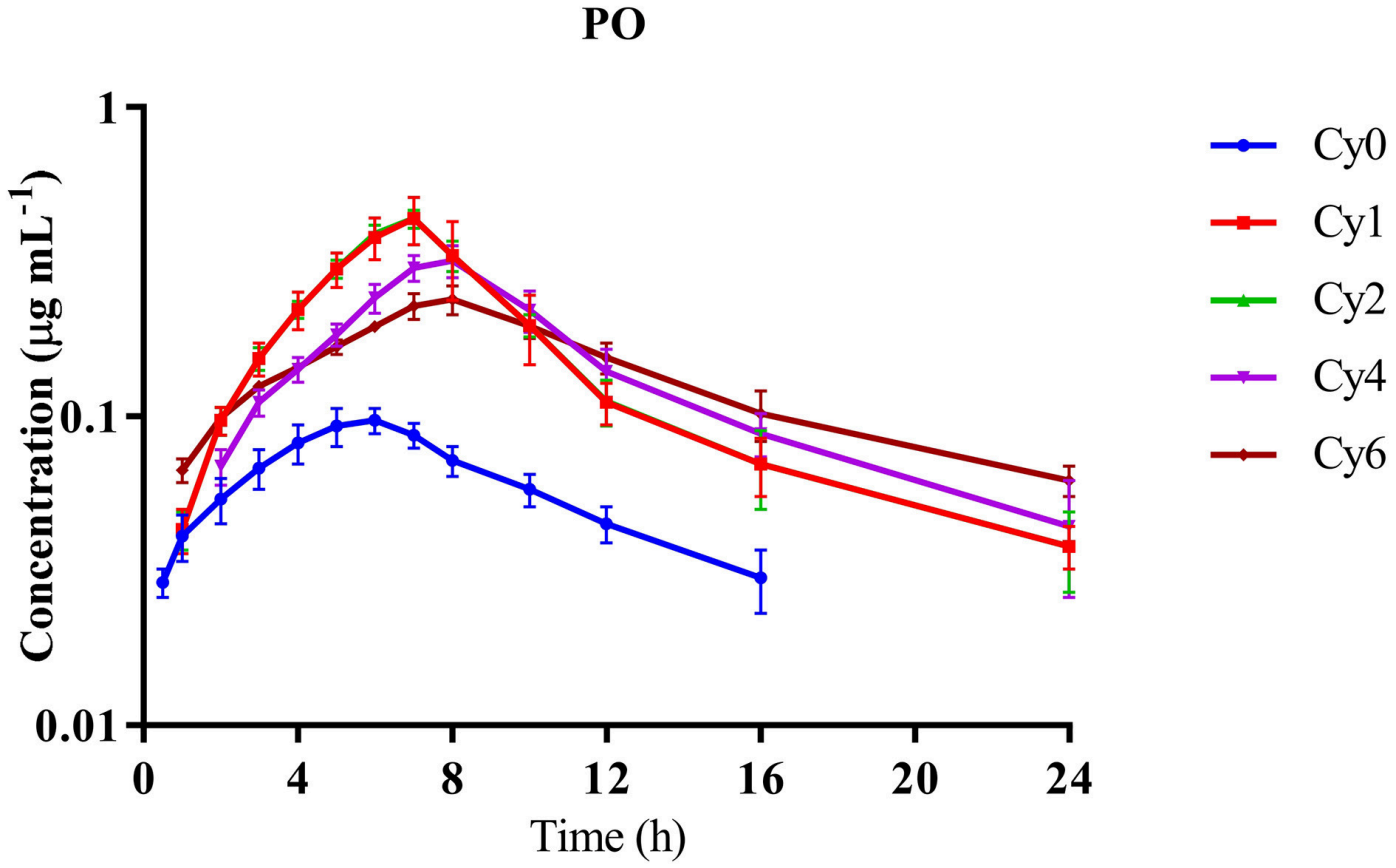
**Mean plasma concentration-time curves of cyadox and its metabolites in dogs after single oral administration through gavage at dose rate 40 mg kg^−1^ b.w. in dogs**. *n* = 6.

**Figure 2:**
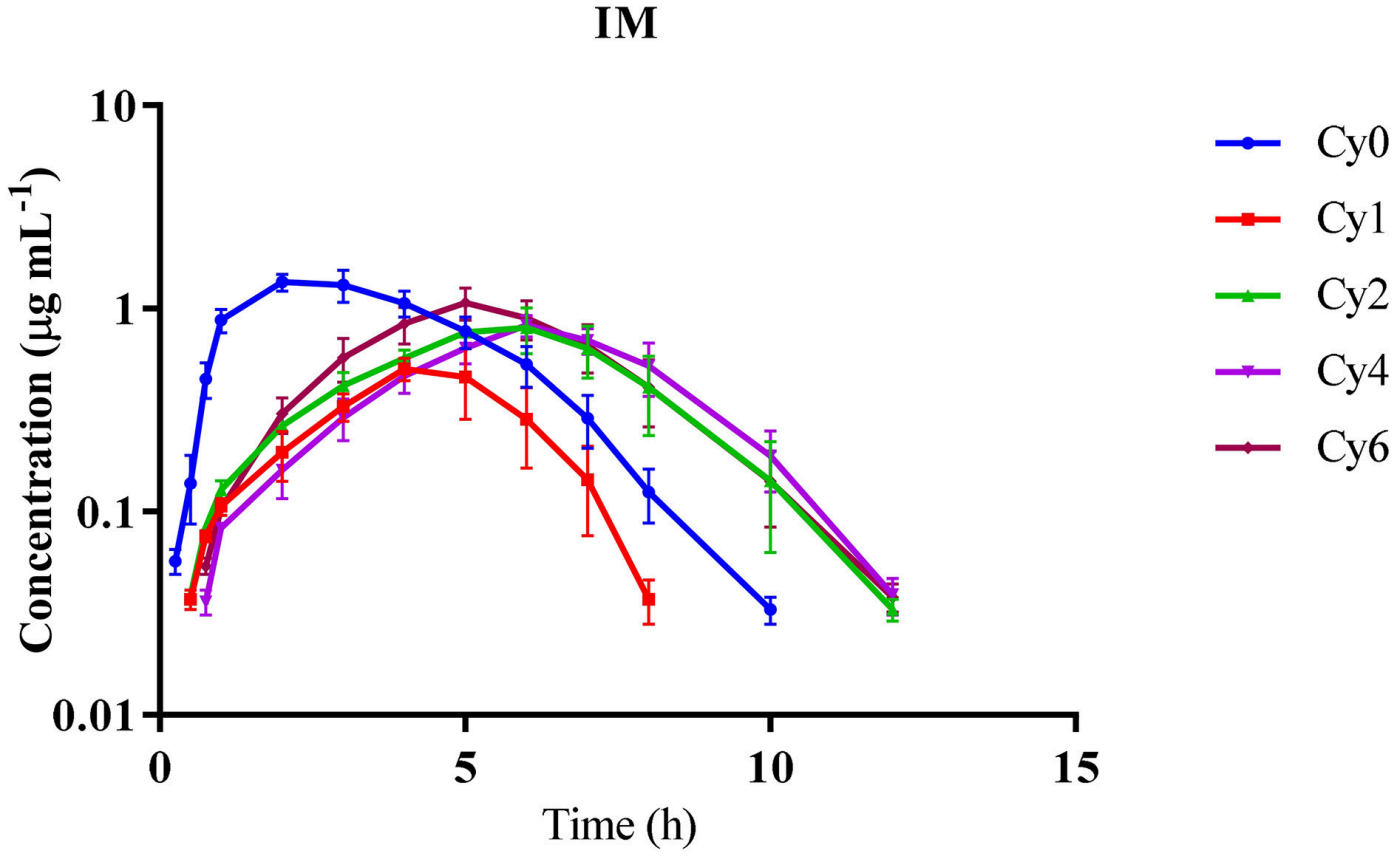
**Mean plasma concentration-time curves of cyadox and its main metabolites after single intramuscular injection in dogs at the dose rate of 10 mg kg^−1^ b.w. *n* = 6**.

**Figure 3:**
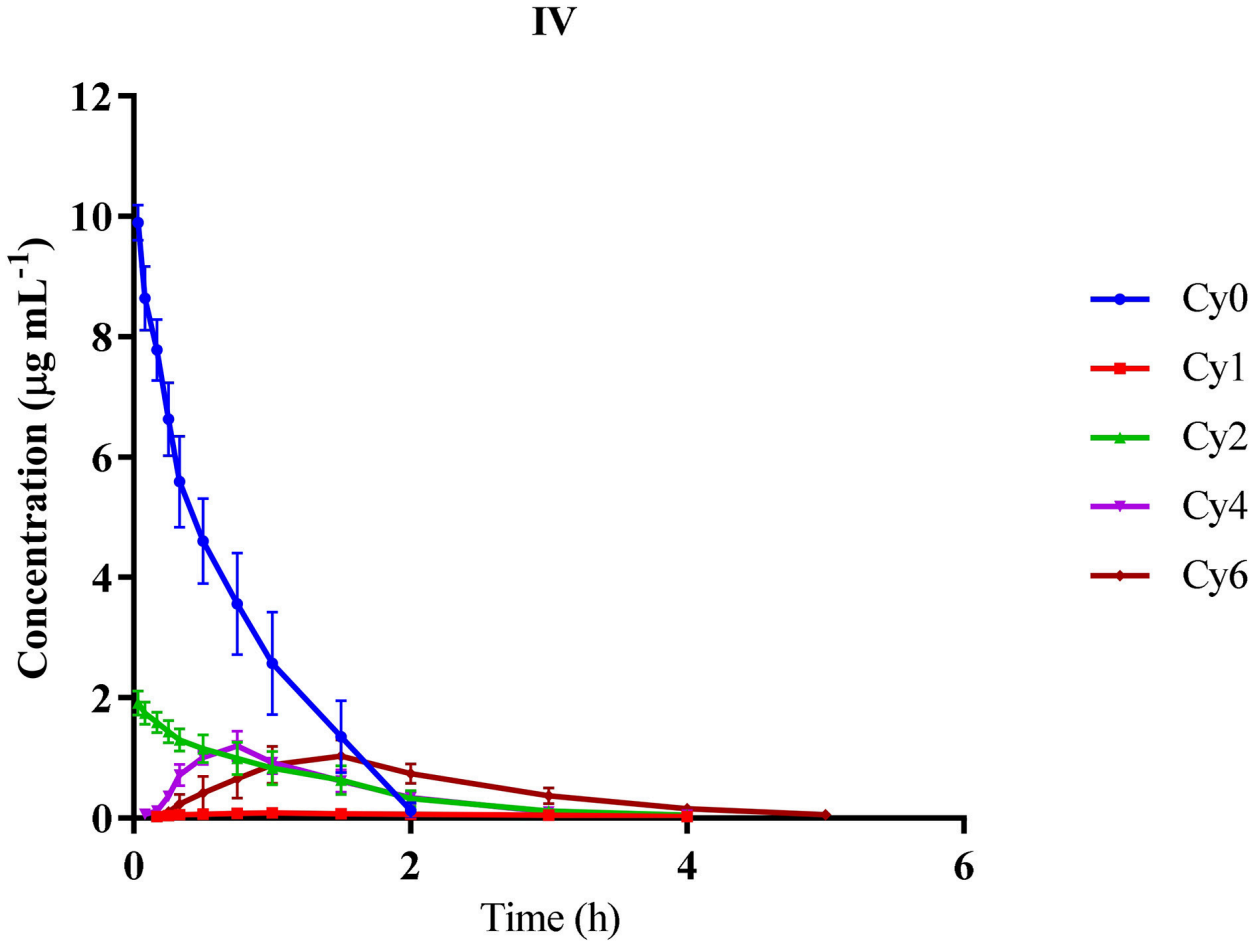
**Mean plasma concentration-time curves of cyadox and its main metabolites after single intravenous injection in dogs at the dose rate of 10 mg kg^−1^ b.w. *n* = 6**.

Plasma pharmacokinetics of Cyx and its main metabolites (Cy1, Cy2, Cy4, and Cy6) was evaluated after PO, IM, or IV administration. The plasma concentration vs. time curves with PO, IM, and IV routes are shown in Figures 1–**3**, respectively. The Cyx concentration in plasma during PO administration reached the maximum level (0.102 ± 0.009 μg mL^−1^) at 5.83 ± 0.75 h after dosing and was below LOQ at 16 h. The maximum concentrations of Cy1, Cy2, Cy4, and Cy6 were higher than parent compound at 7 ± 0.63, 9.33 ± 1, 7.67 ± 0.52, and 7.67 ± 0.52 h, respectively, after dosing. Furthermore, plasma concentrations of Cy1, Cy2, Cy4, and Cy6 reached below LOQ at 24 h after dosing, suggesting that these metabolites are retained in the blood for a longer period than the parent compound, as the terminal half-life (T_1/2_) of Cyx (6.15 ± 0.94 h) was significantly lower than those of Cy1, Cy2, Cy4, and Cy6. Areas under the curve (AUC) of all the metabolites were considerably larger than that of Cyx and oral bioavailability (F) of Cyx was only 4.75 ± 1.39% after PO administration, suggesting lower bioavailability. The volume of distribution (Vz) and body clearance rate (CL) of Cy1, Cy2, Cy4, and Cy6 were lower than that of Cyx (Table 2).

**Table 2:** **Mean plasma pharmacokinetics of cyadox and its metabolites in dogs following oral administration through gavage at dose 40 mg kg^−1^ b.w**.

**Parameters**	**Units**	**Cyx**	**Cy1**	**Cy2**	**Cy4**	**Cy6**
Lz	h^−1^	0.115 ± 0.02	0.089 ± 0.02	0.094 ± 0.03	0.101 ± 0.02	0.085 ± 0.01
T_1/2_	h	6.15 ± 0.94	8.06 ± 1.76	7.97 ± 2.63	7.06 ± 1.34	8.31 ± 1.32
T_max_	h	5.83 ± 0.75	7 ± 0.63	9.33 ± 1	7.67 ± 0.52	7.67 ± 0.52
C_max_	μg mL^−1^	0.102 ± 0.009	0.476 ± 0.04	0.188 ± 0.03	0.336 ± 0.02	0.249 ± 0.02
AUC_0−∞_	h × μg mL^−1^	1.22 ± 0.12	3.89 ± 0.38	3.06 ± 0.28	3.61 ± 0.57	3.85 ± 0.3
AUMC_0−∞_	h × h × μg mL^−1^	13.8 ± 3.29	47.04 ± 8.73	47.96 ± 15.57	48.43 ± 15.42	60.23 ± 10.58
VZ	L kg^−1^	291.35 ± 34.9	119.9 ± 24.3	148.25 ± 38	112.74 ± 7.29	124.1 ± 13.18
CL	L h^−1^ kg^−1^	33.07 ± 3.1	10.37 ± 1.08	13.16 ± 1.26	11.31 ± 1.74	0.085 ± 0.01
MRT_last_	h	7.32 ± 0.43	8.93 ± 0.38	11.68 ± 2.12	10.06 ± 0.5	10.61 ± 0.39
F	%	4.75 ± 1.39				

*Lz, elimination rate constant; T_1/2_, elimination half-life; T_max_, time to reach maximum concentration; C_max_, maximum concentration of drug; AUC_0−∞_, area under plasma concentration-time curve; AUMC_0−∞_, area under first moment curve; VZ, volume of distribution; CL, clearance rate; MRT_last_, mean resident time; F, bioavailability of drug. Data are mean ± SD, n = 6*.

The concentration of Cyx following IM delivery at 2.33 ± 0.52 h was higher than that of its metabolites. AUC and T_1/2_ of Cy1 were lower than the corresponding measurements of Cyx and other main metabolites while Vz (2.3 ± 0.4 L kg^−1^) and CL (1.59 ± 0.154 L h^−1^ kg^−1^) of Cyx were considerably lower, relative to those of its metabolites. Mean resident times (MRT) of Cy1, Cy2, Cy4, and Cy6 were greater than those of the parent metabolite (Table 3). The concentration vs. time curves after IM injection suggested that Cyx reaches the peak level earlier but is quickly eliminated from plasma, compared to metabolites, which showed a swift decrease in concentration but were detectable for up to 12 h after dosing, except Cy1, which was observed for up to 8 h (Figure 2).

**Table 3:** **Mean plasma pharmacokinetic parameters of cyadox and its metabolites in dogs following intramuscular injection at dose 10 mg kg^−1^ b.w**.

**Parameters**	**Units**	**Cyx**	**Cy1**	**Cy2**	**Cy4**	**Cy6**
Lz	1/h	0.702 ± 0.06	0.953 ± 0.15	0.606 ± 0.07	0.621 ± 0.08	0.566 ± 0.09
T_1/2_	h	0.995 ± 0.09	0.741 ± 0.1	1.16 ± 0.15	1.13 ± 0.15	1.25 ± 0.18
T_max_	h	2.33 ± 0.52	4.33 ± 0.52	5.66 ± 0.51	6.17 ± 0.41	5.17 ± 0.4
C_max_	μg mL^−1^	1.48 ± 0.11	0.577 ± 0.1	0.863 ± 0.12	0.853 ± 0.05	1.11 ± 0.2
AUC_0−∞_	h × μg mL^−1^	6.3 ± 0.6	2.08 ± 0.4	4.57 ± 0.85	4.42 ± 0.5	5.45 ± 0.99
AUMC_0−∞_	h × h × μg mL^−1^	23.02 ± 3.27	9.02 ± 2.13	26.21 ± 6.05	27.55 ± 3.61	30.32 ± 5.94
Vz	L kg^−1^	2.3 ± 0.4	5.37 ± 1.46	3.83 ± 1.2	3.76 ± 0.82	3.45 ± 1.06
CL	L h^−1^ kg^−1^	1.59 ± 0.154	4.95 ± 0.9	2.26 ± 0.44	2.29 ± 0.28	1.89 ± 0.37
MRT_last_	h	3.58 ± 0.19	4.22 ± 0.25	5.59 ± 0.32	6.12 ± 0.28	5.45 ± 0.24
F	%	97.28 ± 20.37				

*Lz, elimination rate constant; T_1/2_, elimination half-life; T_max_, time to reach maximum concentration; C_max_, maximum concentration of drug; AUC_0−∞_, area under plasma concentration-time curve; AUMC_0−∞_, area under first moment curve; VZ, volume of distribution; CL, clearance rate; MRT_last_, mean resident time; F, bioavailability of drug. Data are mean ± SD, n = 6*.

The concentrations of Cyx and Cy2 during IV injection were highest at the first time-point (0.033 h) whereas Cy1, Cy4, and Cy6 showed a swift increase followed by decrease in concentration. The T_1/2_ of Cyx (0.344 ± 0.08 h) was markedly lower while AUC of Cyx (6.66 ± 1.21 h × μg mL^−1^) was significantly higher, compared to all the metabolites. Vz and CL of Cyx markedly lower than those of the metabolites, with Cy1 displaying the maximum Vz and CL values. Furthermore, the steady-state volume of distribution (Vss) was the lowest for Cyx (0.873 ± 0.07 L kg^−1^) and highest for Cy1 (100.23 ± 7.12 L kg^−1^). MRT of Cyx was also lower than that of its metabolites (Table 4). We observed a gradual increase in the concentrations of Cy1, Cy4, and Cy6, in contrast to Cyx and Cy2 for which maximum concentrations were observed at the first time-point (Figure 3).

**Table 4:** **Mean plasma pharmacokinetic parameters of cyadox and its metabolites in dogs following intravenous injection at dose 10 mg kg^−1^ b.w**.

**Parameter**	**Units**	**Cyx**	**Cy1**	**Cy2**	**Cy4**	**Cy6**
Lz	1/h	2.09 ± 0.41	0.357 ± 0.03	0.909 ± 0.09	0.944 ± 0.12	0.839 ± 0.1
T_1/2_	h	0.344 ± 0.08	1.95 ± 0.17	0.768 ± 0.07	0.746 ± 0.11	0.836 ± 0.09
T_max_	h	0.033	0.958 ± 0.1	0.033	0.708 ± 0.1	1.42 ± 0.2
C_max_	μg mL^−1^	9.89 ± 0.3	0.089 ± 0.006	1.91 ± 0.2	1.24 ± 0.17	1.08 ± 0.25
AUC_0−∞_	h × μg mL^−1^	6.66 ± 1.21	0.313 ± 0.03	2.2 ± 0.56	1.75 ± 0.34	2.33 ± 0.66
AUMC _0−∞_	h × h × μg mL^−1^	3.92 ± 1.21	0.983 ± 0.17	2.44 ± 0.79	2.4 ± 0.5	4.73 ± 1.28
Vz	L kg^−1^	0.747 ± 0.11	90.35 ± 6.9	5.29 ± 1.24	6.52 ± 2.57	5.62 ± 1.93
CL	L h^−1^kg^−1^	1.55 ± 0.32	32.25 ± 3.41	4.84 ± 1.41	5.93 ± 1.34	4.64 ± 1.51
MRT_last_		0.556 ± 0.07	1.78 ± 0.05	0.981 ± 0.11	1.243 ± 0.05	1.91 ± 0.15
Vss	L kg^−1^	0.873 ± 0.07	100.23 ± 7.12	5.15 ± 1	8.11 ± 1.8	9.57 ± 3.46

*Lz, elimination rate constant; T_1/2_, elimination half-life; T_max_, time to reach maximum concentration; C_max_, maximum concentration of drug; AUC_0−∞_, area under plasma concentration-time curve; AUMC_0−∞_, area under first moment curve; VZ, volume of distribution; CL, clearance rate; MRT_last_, mean resident time; Vss, volume of distribution at steady state. Data are mean ± SD, n = 6*.

### Mass balance in urine and feces

**Figure 4:**
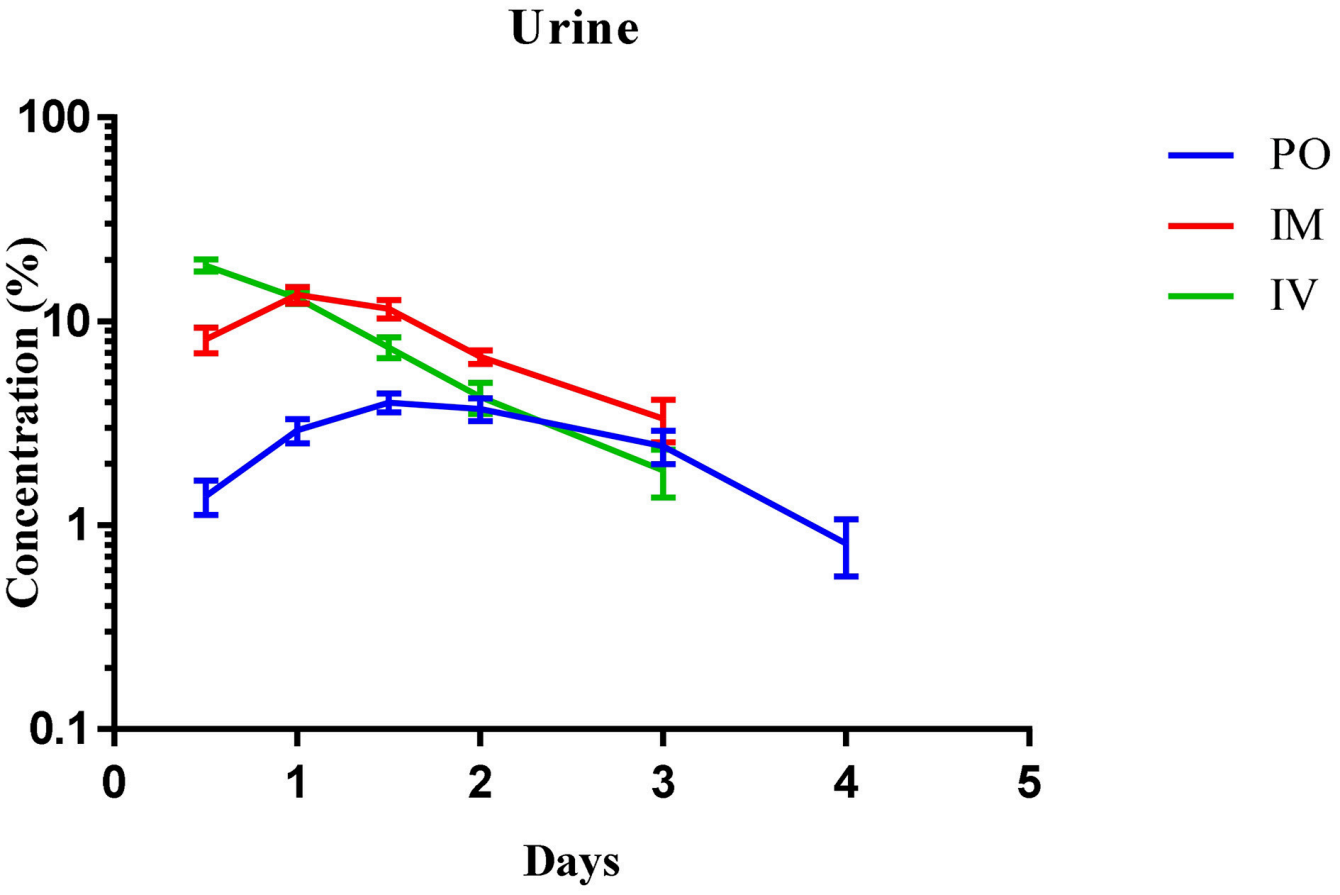
**Mean urine concentration-time curves of cyadox after oral, intramuscular and intravenous routes of administration**. *n* = 6.

**Figure 5:**
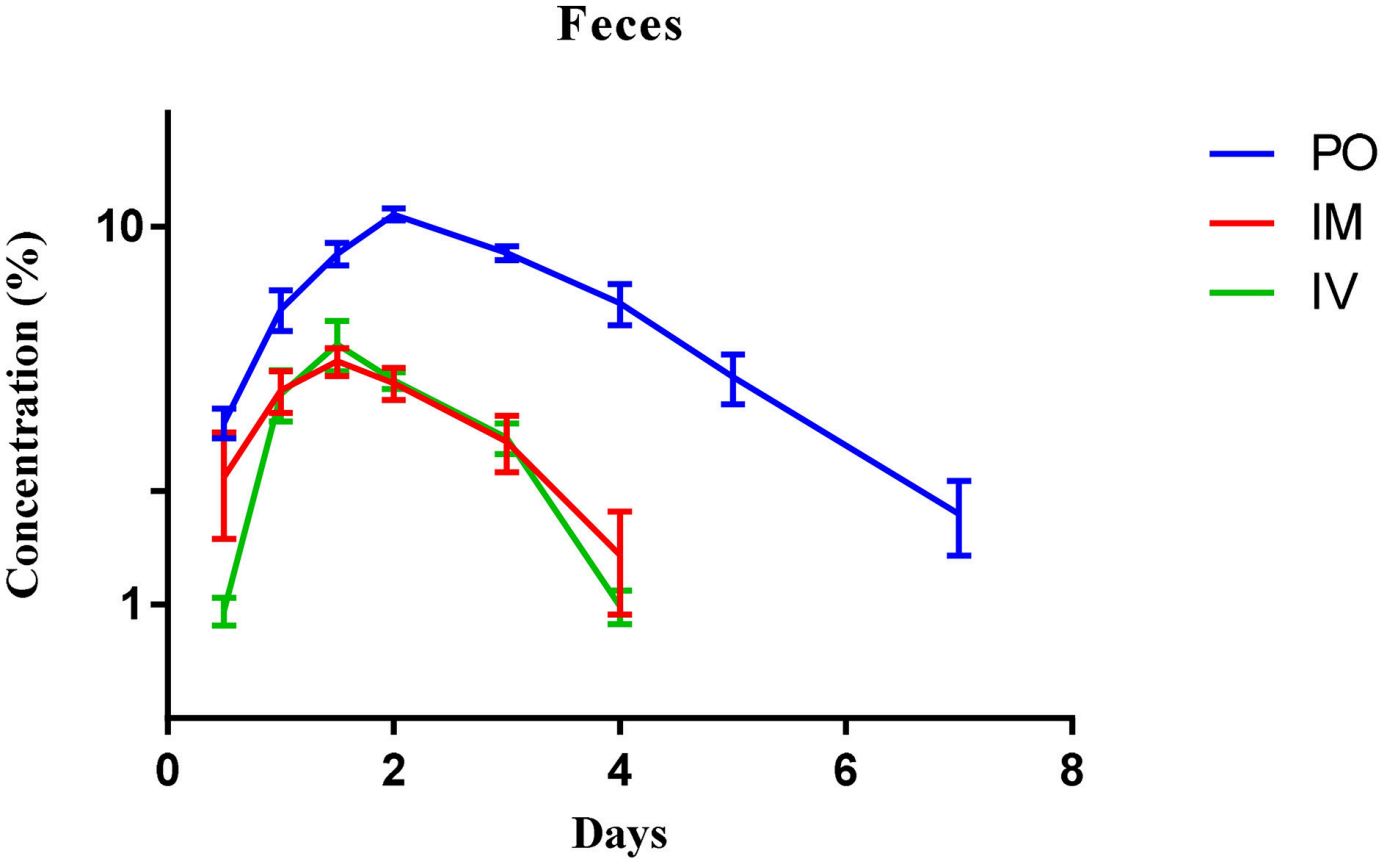
**Mean feces concentration-time curves of cyadox after oral, intramuscular and intravenous routes of administration**. *n* = 6.

Urine and feces concentrations vs. time curves of Cyx after administration via the PO, IM, and IV routes are shown in Figures 4, 5, respectively. The majority of Cyx was excreted through feces upon PO delivery. The consistency and appearance of feces and urine was normal during all collection periods. After PO administration through gavage, the drug was detected for 7 days in feces and 4 days in urine. The maximum concentration in feces (10.78 ± 0.38%) was observed on day 2, while in urine, the concentration (4 ± 0.43%) was highest after 36 h. The cumulative recovery percentage in feces and urine after PO dosing were 48.83 ± 1.99 and 15.3 ± 1.71%, respectively (Table 5).

**Table 5:** **Percentage recovery of cyadox in feces and urine of dogs after oral, intramuscular, and intravenous administrations**.

**Days**	**PO**		**IM**		**IV**	
	**Feces**	**Urine**	**Feces**	**Urine**	**Feces**	**Urine**
0.5	3.02 ± 0.27	1.39 ± 0.27	2.17 ± 0.68	8.14 ± 1.17	0.96 ± 0.08	18.8 ± 1.31
1	6.04 ± 0.75	2.92 ± 0.4	3.68 ± 0.46	13.49 ± 1.23	3.61 ± 0.56	13 ± 0.83
1.5	8.48 ± 0.58	4 ± 0.43	4.40 ± 0.37	11.53 ± 1.19	4.88 ± 0.74	7.45 ± 0.87
2	10.78 ± 0.38	3.73 ± 0.48	3.85 ± 0.38	6.70 ± 0.53	3.92 ± 0.2	4.26 ± 0.74
3	8.52 ± 0.35	2.45 ± 0.46	2.70 ± 0.46	3.34 ± 0.79	2.76 ± 0.26	1.86 ± 0.49
4	6.27 ± 0.78	0.81 ± 0.25	1.35 ± 0.41	ND	0.99 ± 0.098	ND
5	4 ± 0.6	ND	ND	ND	ND	ND
7	1.74 ± 0.39	ND	ND	ND	ND	ND
Sum	48.83 ± 1.99	15.3 ± 1.71	18.15 ± 0.83	43.67 ± 1.19	17.11 ± 1.37	45.37 ± 1.46
Total recovery	64.13 ± 2.43	61.35 ± 1.59	62.48 ± 1.63			

*Data are mean ± SD, n = 6*.

The percentage recovery of Cyx in urine and feces after IM injection was 43.67 ± 1.19 and 18.15 ± 0.83%, respectively. The main route of excretion after IM injection was urine and the drug was detected for up to 4 days in feces. In urine the drug was detected for up to 3 days. The maximum concentration detected at day 1.5 in feces (4.40 ± 0.37%) and after 1 day of injection (13.49 ± 1.23%) in urine (Table 5).

Total drug recovery in feces and urine after IV injection was 17.11 ± 1.37 and 45.37 ± 1.46%, respectively. The concentration of Cyx peaked after 72 h in feces, whereas in urine, the peak level was observed after 12 h of IV injection. However, the drug was recovered up to 4 days in feces and 3 days in urine after IV injection. Elimination was highest in urine at the first time-point and lowest in feces, compared to the PO and IM routes of administration (Table 5).

## Discussion

While a number of studies on tissue depletion, determination methods and toxicity of Cyx in food-producing animals have been reported (Sestakova and Kopanica, 1988; Zhang et al., 2005; Ding et al., 2006b; Huang et al., 2008, 2015), scarce information is available on the pharmacokinetics and metabolism of Cyx administered via different routes. In view of the relatively low toxicity of Cyx and strong antibacterial effects, this study was designed to evaluate the pharmacokinetics and metabolism of Cyx and four of its major metabolites after administration via PO, IM, and IV routes in beagle dogs. The PO dose was selected on the basis of a previous study conducted in pigs (Zhao et al., 2013), whereas IM and IV doses were established based on earlier pharmacokinetic studies on mequindox, a well-known group member of the Cyx family (Liu et al., 2011; Ding et al., 2012). Cy1, Cy2, Cy4, and Cy6 were previously identified as major metabolites of Cyx in food-producing animals (Zhao et al., 2013; Huang et al., 2015), and therefore selected for investigation in the current study. A highly selective and sensitive UV-HPLC method allowing the simultaneous determination of Cyx and its major metabolites in dog plasma, feces and urine was utilized for comprehensive evaluation of metabolism and pharmacokinetic profiles of Cyx in dogs.

The recovery rates of all five analytes in plasma of dog were >80% using MeOH as the extraction solvent. After testing various solvents alone or in combination for extraction of analytes in urine and feces, best recovery (>70%) was achieved in the presence of 1% metaphosphoric acid at a MeOH/ACN/water ratio of 50:20:30 (v/v/v). Oasis HLB was superior to other cartridges in terms of good recovery (>80% in plasma, >70% in urine and feces) and low matrix interference. For the elution step, ACN and MeOH were used. Ultimately, MeOH was established as the best choice due to its good extraction efficiency.

In general, pharmacokinetic parameters differ according to species, drug formulation, body condition, route of administration, age, gender, and physiological status of animals, all of which contribute to differences in drug efficacy (Canga et al., 2009). In our study, the maximum concentration (C_max_) of Cyx in plasma was 0.102 ± 0.009 μg mL^−1^ at 5.83 ± 0.75 h following PO administration, implying that Cyx is quickly metabolized to its metabolites. Compared to the parent metabolite Cy1, Cy2, Cy4, and Cy6 showed higher concentrations and remained in plasma for longer time-periods (Table 2). The highest concentration of Cyx observed following delivery through IM or IV routes indicates that Cyx is partly metabolized in the gastrointestinal tract (GIT) before absorption due to the presence of certain microflora or enzymes to generate metabolites. Our results were in accordance with data from previous studies conducted in pigs (Zhao et al., 2013). Other members of this group, i.e., quinocetone and mequindox exhibit the same type of response in pigs and chickens when administered IV, IM, or orally (Zhong et al., 2011; Ding et al., 2012; Li et al., 2012). The lower AUC ratio of Cyx compared to its metabolites upon administration through PO route, relative to IM or IV injection, also supports the theory that this drug is quickly metabolized in the GIT and converted to its metabolites. Other recent *in vitro* and *in vivo* studies have validated that GIT is the main metabolic pathway of this drug (Liu et al., 2009; Xu et al., 2011). The terminal half-lives of Cyx when administered orally through gavage and after IM and IV injections revealed that Cyx is more rapidly eliminated from dog plasma than its major metabolites. In an earlier study, the terminal half-lives of Cyx in pig after PO and IV administration were 4.77 h (40 mg kg^−1^ b.w.) and 0.93 h (1 mg kg^−1^ b.w.), respectively (Zhao et al., 2013). The discrepancies in values obtained with both studies may be attributable to species differences. Further C_max_ of Cyx after PO, IM, and IV injections revealed low amount of Cyx in plasma after PO administration. Higher AUC and longer elimination half-lives were detected for all major metabolites (Cy1, Cy2, Cy4, and Cy6), compared to Cyx, with all administration routes in dog plasma, implying that the majority of Cyx is transformed into its metabolites, which possess good stability in plasma. Similar results were observed during pharmacokinetic characterization of mequindox and quinocetone, well-known members of this group (Ding et al., 2012). Therefore, close attention should be paid to these metabolites when identifying residue markers of Cyx in animal tissues. The AUC and MRT values of all metabolites administered via different routes at different doses indicate that Cyx is swiftly absorbed and transformed into metabolites, which remain in the blood for a long time. Furthermore, the concentration vs. time curves obtained after all three routes of administration revealed a rapid increase, followed by gradual decrease in the concentration of Cyx and its metabolites, except in the case of Cyx and Cy2 during IV route of administration, which showed the highest concentrations at the first time-point (Figures 1–3). This finding may be due to the reason that deoxygenation is the initial and primary metabolic pathway of QdNOs, including Cyx. Thus, Cy2 in blood samples was quickly present via the IV route. The poor bioavailability (4.75 ± 1.39%) of Cyx and higher C_max_ values of its metabolites after PO administration indicate that Cyx has an intestinal first-pass effect in dogs. In another study the bioavailability of Cyx was determined as 2.75% in pig, while among the other group members, the bio-availability of mequindox in chicken, pigs and rats was evaluated as 17, 26, and 37%, respectively (Liu et al., 2011; Ding et al., 2012; Li et al., 2012). These findings suggest that the oral bioavailability of Cyx is low, compared to its congeners. To endorse this hypothesis, development of an intestinal and vascular access port (IVAP) may be an effective approach for validating its livery or intestinal first pass effect in animals. Additionally, the low concentrations of Cyx in plasma observed after PO delivery suggests that this route is suitable for intestinal infections while the IM route may be the choice for systemic infections, as higher concentration of Cyx was detected in the circulation than the minimum inhibitory concentration of certain pathogenic bacterial species (Fan et al., 2000; Huang et al., 2002).

Cyx is extensively metabolized in animal body involving N-O group reduction, C = N cleavage, hydrogenation and hydrolysis on the side chain. A number of *in vitro* and *in vivo* experiments have revealed that Cyx can be metabolized through both enzymatic and non-enzymatic pathways in both liver and GIT (Zheng et al., 2011). Firstly, Cyx is quickly metabolized into Cy1 and Cy2 through the process of deoxidation. Subsequently, Cy1 and Cy2 are further deoxidized to form Cy4, which converted into Cy6 through the process of hydrogenation (Xu et al., 2011). In the current study, Cyx was excreted swiftly after all three dosage routes. Fecal excretion was the major route of elimination in dog following PO administration and renal excretion established as the minor route. These results indicate that most of the drug excreted in feces is not absorbed and thus excreted in bile. Analogous findings have recently been reported following PO administration of Cyx in rats, pigs, chickens, and carp (Huang et al., 2015). In view of the finding that Cyx is extensively metabolized by microflora of GIT (Xu et al., 2011), the low amount of Cyx detected in dogs, compared to its metabolites, may indicate similar extensive metabolization by microflora of GIT in these animals. Cyx was detected up to 7 days in dog feces and up to 4 days in urine after PO administration (Table 5). The maximum concentration in feces was detected on day 2 and in urine after 72 h of PO delivery, suggesting rapid elimination from the body. More than 15 metabolites have been identified in animal bodies in earlier studies (Huang et al., 2015). The total percentage recovery of Cyx and its metabolites in feces and urine was calculated as 48.83 ± 1.99% and 15.3 ± 1.71%, respectively, suggesting that Cy1, Cy2, Cy4, and Cy6 are the main metabolites in dog body comprising almost 64% of the drug excreted through urine and feces. Progressive increase and a subsequent steady decrease in drug concentrations in both urine and feces was also observed (Figures 4, 5).

The fate of Cyx after IM and IV injection was determined for the first time in this study. Total recovery rates via IM and IV administration were 43.67 ± 1.19 and 45.37 ± 1.46% in urine and 18.76 ± 0.9 and 17.11 ± 1.37% in feces, respectively, suggesting that urine is the major route of excretion using these two modes of delivery, with a lower amount detected in feces (Table 5). This finding may be attributed to the fact that most of the drug does not enter the GIT when injected via the IV or IM route, and is excreted from the body through renal clearance. Earlier studies have reported that Cyx is extensively metabolized by pig, chicken and rat liver microsomes (Liu et al., 2009). The high recovery of Cyx and its main metabolites in urine indicates that Cyx is comprehensively metabolized by dog liver microsomes. The maximum percentage of drug following administration through IV and IM routes was detected in urine after 12 h and on day 1 and in feces on day 1.5, with a total detection period of 4 days in feces and 3 days in urine. Moreover, the concentration vs. time curves suggested a gradual increase and subsequent swift decrease of Cyx in feces with all three routes of administration, with high elimination times via PO, compared to IM and IV delivery (Figure 4). In urine, the concentration of Cyx was maximal after 12 h upon IV injection, following which recovery decreased gradually, with detection up to 3 days. Moreover, recovery was higher in the first collection period after IM injection, compared to PO administration (Figure 5). Our findings suggest that Cyx injected via IM and IV routes is rapidly distributed, undergoes renal clearance evading the first-pass effect of GIT, and is extensively metabolized by liver microsomes. However, following IM injection, the concentration was highest on day 1, indicating that Cyx enters the circulation progressively, and the drug remained in the body for up to 3 days.

## Conclusion

In worldwide, Cyx is being developed as a replacer of its congeners because of low toxicity and strong antibacterial effects in animals, compared to other QdNO drugs. It could be the drug of choice in canine because they are more prone to toxic effects against certain drugs which make a narrow range of choices for them. The dog physiology is similar to other mammals which could also help for risk assessment and safety evaluation. This study provides a significant information on pharmacokinetic and metabolism profiles of Cyx and its major metabolites through different routes of administration which will not only improve our understanding about pharmacology and toxicology of Cyx, but also the safety evaluation and clinical use in mammals.

## Ethic statement

The use of dogs in this study was according to Animal Experimental Ethical Inspection of Laboratory Animal Center, Huazhong Agricultural University, Wuhan, China HZAUSW-2016-006. All efforts were made to minimize the suffering of the animals.

## Author contributions

Conceived and designed the experiments: AS, LH, SX, ZY. Performed the experiments: AS, MS, ZI, HH, MI, SX. Analyzed the data: AS, YT, ZL. Contributed reagents/materials/analysis tools: WQ, YP, DC, ZY. Wrote the paper: AS, SX, LH, ZY.

## Funding

This work was supported by National Natural Science Foundation of China (31302140) and Veterinary Drug Residue Warning Technology Import Cooperation of Livestock and Poultry Products (2014-S12).

### Conflict of interest statement

The authors declare that the research was conducted in the absence of any commercial or financial relationships that could be construed as a potential conflict of interest. The reviewer JF and handling Editor declared their shared affiliation, and the handling Editor states that the process nevertheless met the standards of a fair and objective review.
